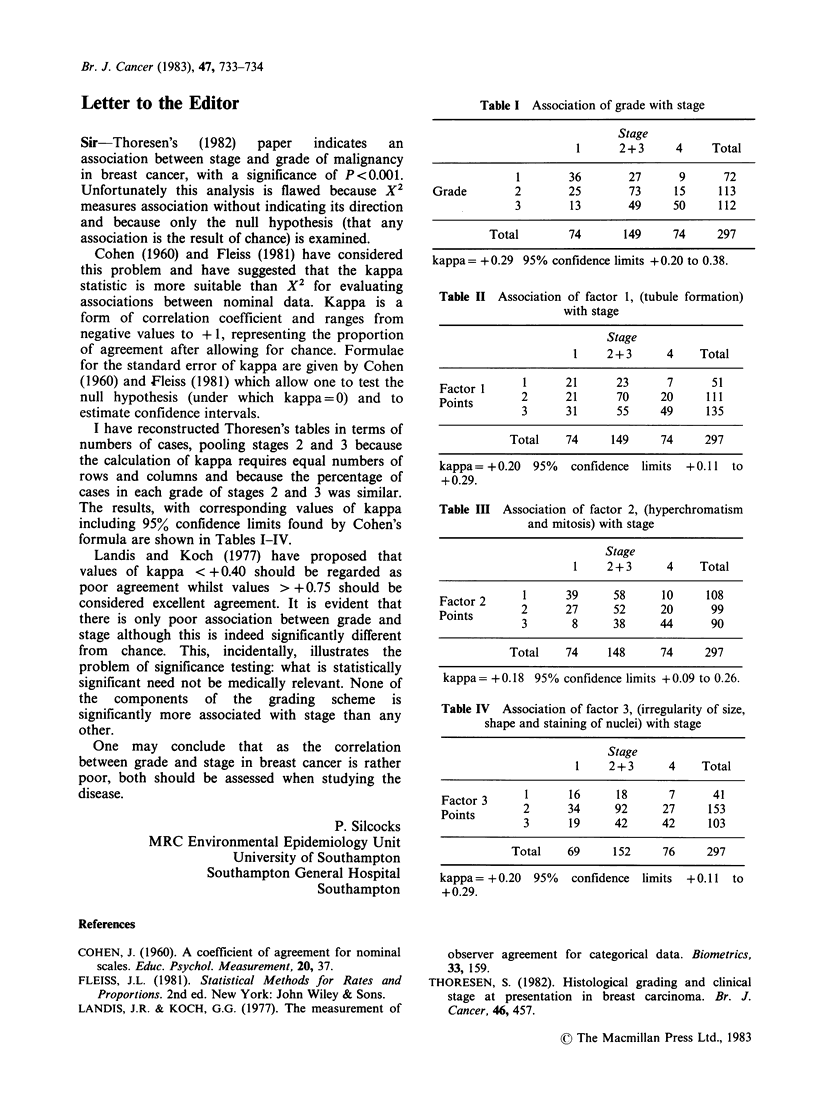# Staging and grading in breast cancer.

**DOI:** 10.1038/bjc.1983.114

**Published:** 1983-05

**Authors:** P. Silcocks


*Br. J. Cancer* (1983), **47**, 733–734

### Letter to the Editor

**Sir**—Thoresen's (1982) paper indicates an association between stage and grade of malignancy in breast cancer, with a significance of $P<0.001$. Unfortunately this analysis is flawed because $X^2$ measures association without indicating its direction and because only the null hypothesis (that any association is the result of chance) is examined.

Cohen (1960) and Fleiss (1981) have considered this problem and have suggested that the kappa statistic is more suitable than $X^2$ for evaluating associations between nominal data. Kappa is a form of correlation coefficient and ranges from negative values to $+1$, representing the proportion of agreement after allowing for chance. Formulae for the standard error of kappa are given by Cohen (1960) and Fleiss (1981) which allow one to test the null hypothesis (under which kappa $=0$) and to estimate confidence intervals.

I have reconstructed Thoresen's tables in terms of numbers of cases, pooling stages 2 and 3 because the calculation of kappa requires equal numbers of rows and columns and because the percentage of cases in each grade of stages 2 and 3 was similar. The results, with corresponding values of kappa including 95% confidence limits found by Cohen's formula are shown in Tables I–IV.

Landis and Koch (1977) have proposed that values of kappa $<+0.40$ should be regarded as poor agreement whilst values $>+0.75$ should be considered excellent agreement. It is evident that there is only poor association between grade and stage although this is indeed significantly different from chance. This, incidentally, illustrates the problem of significance testing: what is statistically significant need not be medically relevant. None of the components of the grading scheme is significantly more associated with stage than any other.

One may conclude that as the correlation between grade and stage in breast cancer is rather poor, both should be assessed when studying the disease.

P. Silcocks
MRC Environmental Epidemiology Unit
University of Southampton
Southampton General Hospital
Southampton

**Table I** Association of grade with stage

| | | Stage 1 | Stage 2+3 | Stage 4 | Total |
|---|---|---|---|---|---|
| Grade | 1 | 36 | 27 | 9 | 72 |
| | 2 | 25 | 73 | 15 | 113 |
| | 3 | 13 | 49 | 50 | 112 |
| | Total | 74 | 149 | 74 | 297 |

kappa = +0.29 95% confidence limits +0.20 to 0.38.

**Table II** Association of factor 1, (tubule formation) with stage

| | | Stage 1 | Stage 2+3 | Stage 4 | Total |
|---|---|---|---|---|---|
| Factor 1 Points | 1 | 21 | 23 | 7 | 51 |
| | 2 | 21 | 70 | 20 | 111 |
| | 3 | 31 | 55 | 49 | 135 |
| | Total | 74 | 149 | 74 | 297 |

kappa = +0.20 95% confidence limits +0.11 to +0.29.

**Table III** Association of factor 2, (hyperchromatism and mitosis) with stage

| | | Stage 1 | Stage 2+3 | Stage 4 | Total |
|---|---|---|---|---|---|
| Factor 2 Points | 1 | 39 | 58 | 10 | 108 |
| | 2 | 27 | 52 | 20 | 99 |
| | 3 | 8 | 38 | 44 | 90 |
| | Total | 74 | 148 | 74 | 297 |

kappa = +0.18 95% confidence limits +0.09 to 0.26.

**Table IV** Association of factor 3, (irregularity of size, shape and staining of nuclei) with stage

| | | Stage 1 | Stage 2+3 | Stage 4 | Total |
|---|---|---|---|---|---|
| Factor 3 Points | 1 | 16 | 18 | 7 | 41 |
| | 2 | 34 | 92 | 27 | 153 |
| | 3 | 19 | 42 | 42 | 103 |
| | Total | 69 | 152 | 76 | 297 |

kappa = +0.20 95% confidence limits +0.11 to +0.29.

### References

COHEN, J. (1960). A coefficient of agreement for nominal scales. *Educ. Psychol. Measurement*, **20**, 37.

FLEISS, J.L. (1981). *Statistical Methods for Rates and Proportions*. 2nd ed. New York: John Wiley & Sons.

LANDIS, J.R. & KOCH, G.G. (1977). The measurement of observer agreement for categorical data. *Biometrics*, **33**, 159.

THORESEN, S. (1982). Histological grading and clinical stage at presentation in breast carcinoma. *Br. J. Cancer*, **46**, 457.

© The Macmillan Press Ltd., 1983